# Supplementary material for: Prevalence of Antibodies to SARS-CoV-2 Following Natural Infection and Vaccination in Irish Hospital Healthcare Workers: Changing Epidemiology as the Pandemic Progresses
Source: Front Med (Lausanne). 2022 Feb 4;8:758118. doi: 10.3389/fmed.2021.758118 (PMC8854655; doi:10.3389/fmed.2021.758118)
Supplement: Supplementary file 1 [file Presentation_1.pdf]

Annex

Table A Characteristics of HCWs with SARS-CoV-2 seropositivity (n=898), by hospital, April 2021

**Participant characteristics**		**Hospital 1**		**Hospital 2**		**Both Hospitals**	
		**n**	**%**	**n**	**%**	**n**	**%**
**Overall**		623	21%	275	13%	898	18%
**Median age (IQR)**		39 (29-49)		35 (27-44)		38 (29-48)	
**Age groups (years)**	18-29	159	26%	90	33%	249	28%
	30-39	154	25%	84	31%	238	27%
	40-49	157	25%	51	19%	208	23%
	50-59	119	19%	39	14%	158	18%
	Over 60	34	5.5%	11	4.0%	45	5.0%
**Sex**	Female	471	76%	198	72%	669	74%
	Male	152	24%	77	28%	229	26%
**Ethnicity**	Irish	401	64%	194	71%	595	66%
	Any other white background	56	9.0%	38	14%	94	10%
	Asian background	122	20%	26	9.5%	148	16%
	African/other black background	29	4.7%	10	3.6%	39	4.3%
	Other	15	2.4%	7	2.5%	22	2.4%
**Country of birth**	Ireland	386	62%	181	66%	567	63%
	United Kingdom	25	4.0%	19	6.9%	44	4.9%
	India	60	10%	16	5.8%	76	8.5%
	Philippines	53	8.5%	1	0.4%	54	6.0%
	Poland	8	1.3%	12	4.4%	20	2.2%
	USA	4	0.6%	7	2.5%	11	1.2%
	Romania	12	1.9%	3	1.1%	15	1.7%
	Nigeria	18	2.9%	1	0.4%	19	2.1%
	Other	57	9.1%	35	13%	92	10%
**Education**	Primary	7	1.1%	0	0.0%	7	0.8%
	Secondary	105	17%	28	10%	133	15%
	Third level	301	48%	133	48%	434	48%
	Post-graduate	210	34%	114	41%	324	36%
**Role**	Admin	64	10%	21	7.6%	85	9.5%
	Medical/dental	55	8.8%	61	22%	116	13%
	Nursing/ midwifery	281	45%	114	41%	395	44%
	Allied health	89	14%	29	11%	118	13%
	General support	60	10%	21	7.6%	81	9.0%
	Health care assistant	70	11%	23	8.4%	93	10%
	Other	4	0.6%	6	2.2%	10	1.1%
**Lives with**	Alone	42	6.7%	19	6.9%	61	6.8%
	With others	578	92.8%	256	93%	834	92.9%
	Missing	3	0.5%	0	0.0%	3	0.3%
**Lives with HCW**	Yes	234	38%	106	39%	340	38%
	No	376	60%	164	60%	540	60%
	Missing	13	2.1%	5	1.8%	18	2.0%
**Daily contact with COVID-19 patients**	Contact with COVID-19 patients	196	31%	69	25%	265	30%
	Contact with patients without COVID-19	293	47%	170	62%	463	52%
	No patient contact	134	22%	36	13%	170	19%

**Previous COVID-19 symptoms**	No symptoms	121	19%	48	17%	169	19%
	Had symptoms	502	81%	227	83%	729	81%
	Missing	0	0.0%	0	0.0%	0	0.0%
**Severity of symptoms**	No symptoms	121	19%	48	17%	169	19%
	Mild symptoms	228	37%	107	39%	335	37%
	Significant symptoms	262	42%	102	37%	364	41%
	Severe (hospitalised)	12	1.9%	18	6.5%	30	3.3%
	Missing	0	0.0%	0	0.0%	0	0.0%
**Previous positive COVID-19 PCR test**	No	190	30%	45	16%	235	26%
	Yes	433	70%	230	84%	663	74%
**Symptoms at time of previous positive PCR test**	No	51	12%	36	16%	87	13%
	Yes	382	88%	194	84%	576	87%
**Severity of symptoms at time of PCR test**	No symptoms	51	12%	36	16%	87	13%
	Mild symptoms	143	33%	83	36%	226	34%
	Significant symptoms	227	52%	93	40%	320	48%
	Severe (hospitalised)	12	2.8%	18	7.8%	30	4.5%
	Missing	0	0.0%	0	0.0%	0	0.0%

Table 2c Prevalence of SARS-CoV-2 seropositivity by participant characteristics, Hospital 1, April 2021

**Participant characteristics**		Total	SARS-CoV-2 seropositive		
		**N**	**n**	**% (95% CI)**	**p-value***
**Overall**		2945	623	21 (20 - 23)	-
**Age groups (years)**	18-29	653	159	24 (21 - 28)	0.184
	30-39	765	154	20 (17 - 23)	
	40-49	811	157	19 (17 - 22)	
	50-59	565	119	21 (18 - 25)	
	Over 60	151	34	23 (16 - 30)	
**Sex**	Female	2,278	471	21 (19 - 22)	0.240
	Male	667	152	23 (20 - 26)	
**Ethnicity**	Irish	2,091	401	19 (18 - 21)	<0.001
	Any other white background	257	56	22 (17 - 27)	
	Asian background	470	122	26 (22 - 30)	
	African or any other black background	69	29	42 (30 - 55)	
	Other	58	15	26 (15 - 39)	
**Country of birth**	Ireland	2,025	386	19 (17 - 21)	0.001
	United Kingdom	134	25	19 (12 - 26)	
	India	225	60	27 (21 - 33)	
	Philippines	198	53	27 (21 - 34)	
	Poland	26	8	31 (14 - 52)	
	USA	21	4	19 (5.4 - 42)	
	Romania	40	12	30 (17 - 47)	
	Nigeria	25	18	72 (51 – 88)	
	Other	251	57	23 (18 - 28)	
**Education**	Primary	20	7	35 (15 - 59)	<0.001
	Secondary	409	105	26 (22 - 30)	
	Third level	1,280	301	24 (21 - 26)	
	Post-graduate	1,236	210	17 (15 - 19)	
**Role**	Admin	403	64	16 (12 - 20)	<0.001
	Medical/dental	357	55	15 (12 - 20)	
	Nursing/ midwifery	1097	281	26 (23 - 28)	
	Allied health	612	89	15 (12 - 18)	
	General support	243	60	25 (19 - 31)	
	Health care assistant	179	70	39 (32 - 47)	
	Other	54	4	7.4 (2.1 - 18)	
**Lives with**	Alone	270	42	16 (11 - 20)	0.019
	With others	2,667	578	22 (20 - 23)	
	Missing	8	3	38 (8.5 - 76)	
**Lives with HCW**	Yes	928	234	25 (22 - 28)	<.001
	No	1,964	376	19 (17 - 21)	
	Missing	53	13	25 (14 - 38)	

*Calculated using the Chi-square test

Table 2d Prevalence of SARS-CoV-2 seropositivity by COVID-19 related characteristics, Hospital 1, April 2021

**COVID-19 related characteristics**		Total	SARS-CoV-2 seropositive		
		**N**	**n**	**% (95% CI)**	**p-value***
**Daily contact with COVID-19 patients**	Contact with COVID-19 patients	726	196	27 (24 - 30)	<0.001
	Contact with patients without COVID-	1,362	293	22 (19 - 24)	
	No patient contact	857	134	16 (13 - 18)	
**Previous COVID-19 symptoms**	No symptoms	1571	121	7.7 (6.4 - 9.1)	<0.001
	Had symptoms	1374	502	37 (34 - 39)	
	Missing	0	0	-	
**Severity of symptoms**	No symptoms	1571	121	7.7 (6.4 - 9.1)	<0.001
	Mild symptoms	932	228	24 (22 - 27)	
	Significant symptoms	420	262	62 (58 - 67)	
	Severe (hospitalised)	21	12	57 (34 - 78)	
	Missing	1	0	-	
**Previous positive COVID-19 PCR test**	No	2427	190	7.8 (6.8 - 9.0)	<0.001
	Yes	518	433	84 (80 - 87)	
**Symptoms at time of previous positive PCR**	No	95	51	54 (43 - 64)	<0.001
	Yes	423	382	90.3 (87 - 93.0)	
**Severity of symptoms at time of PCR test**	No symptoms	95	51	54 (43 - 64)	<0.001
	Mild symptoms	162	143	88 (82 - 92.8)	
	Significant symptoms	248	227	91.5 (87 - 94.7)	
	Severe (hospitalised)	12	12	100 (74 - 100)	
	Missing	1	0	-	

*Calculated using the Chi-square test

Table 2e Prevalence of SARS-CoV-2 seropositivity by participant characteristics, Hospital 2, April 2021

Participant characteristics		Total	SARS-CoV-2 seropositive		
		**N**	**n**	**% (95% CI)**	**p-value***
**Overall**		2140	275	13 (11 - 14)	-
**Age groups (years)**	18-29	455	90	20 (16 - 24)	<0.001
	30-39	565	84	15 (12 - 18)	
	40-49	603	51	8.5 (6.4 - 11)	
	50-59	386	39	10 (7.3 - 14)	
	Over 60	131	11	8.4 (4.3 - 15)	
**Sex**	Female	1,681	198	12 (10 - 13)	0.005
	Male	459	77	17 (13 - 21)	
**Ethnicity**	Irish	1,707	194	11 (10 - 13)	0.002
	Any other white background	219	38	17 (13 - 23)	
	Asian background	129	26	20 (14 - 28)	
	African or any other black background	48	10	21 (10 - 35)	
	Other	37	7	19 (8.0 - 35)	
**Country of birth**	Ireland	1605	181	11 (10 - 13)	<0.001
	United Kingdom	161	19	12 (7.3 - 18)	
	India	68	16	24 (14 - 35)	
	Poland	59	12	20 (11 - 33)	
	USA	34	7	21 (8.7 - 38)	
	Philippines	16	1	6.3 (0.2 - 30)	
	Nigeria	10	1	10 (0.3 - 45)	
	Romania	5	3	60 (14 - 95)	
	Other	182	39	19 (13 - 36)	
**Education**	Primary	2	0	-	0.485
	Secondary	200	28	14 (10 - 20)	
	Third level	964	133	14 (12 - 16)	
	Post-graduate	974	114	12 (10 - 14)	
**Role**	Admin	273	21	7.7 (4.8 - 12)	<0.001
	Medical/dental	356	61	17 (13 - 21)	
	Nursing/ midwifery	794	114	14 (12 - 17)	
	Allied health	432	29	6.7 (4.5 - 9.5)	
	General support	122	21	17 (11 - 25)	
	Health care assistant	112	23	21 (13 - 29)	
	Other	51	6	12 (4.4 - 24)	
**Lives with**	Alone	194	19	10 (6.0 - 15)	0.180
	With others	1,943	256	13 (12 - 15)	
	Missing	3	0	-	
**Lives with HCW**	Yes	643	106	16 (14 - 20)	0.001
	No	1448	164	11 (10 - 13)	
	Missing	49	5	10 (3.4 - 22)	

*Calculated using the Chi-square test

Table 2f Prevalence of SARS-CoV-2 seropositivity by COVID-19 characteristics, Hospital 2, April 2021

**COVID-19 related characteristics**		Total	SARS-CoV-2 seropositive		
		**N**	**n**	**% (95% CI)**	**p-value***
**Daily contact with COVID-19 patients**	Contact with COVID-19 patients	410	69	17 (13 - 21)	<0.001
	Contact with patients without COVID-19	1,138	170	15 (13 - 17)	
	No patient contact	592	36	6.1 (4.3 - 8.3)	
**Previous COVID-19 symptoms**	No symptoms	1342	48	3.6 (2.6 - 4.7)	<0.001
	Had symptoms	797	227	28 (25 - 32)	
	Missing	1	0	-	
**Severity of symptoms**	No symptoms	1342	48	3.6 (2.6 - 4.7)	<0.001
	Mild symptoms	570	107	19 (16 - 22)	
	Significant symptoms	201	102	51 (48 - 58)	
	Severe (hospitalised)	26	18	69 (44 - 86)	
	Missing	0	0	-	
**Previous positive COVID-19 PCR**	No	1846	45	2.4 (1.8 - 3.2)	<0.001
	Yes	294	230	78 (73 - 83)	
**Symptoms at time of previous positive PCR**	No	77	36	47 (35 - 58)	<0.001
	Yes	217	194	89 (85 - 93.2)	
**Severity of symptoms at time of PCR test**	No symptoms	77	36	47 (35 - 58)	<0.001
	Mild symptoms	94	83	88 (80 - 94.0)	
	Significant symptoms	103	93	90.3 (83 - 95.2)	
	Severe (hospitalised)	20	18	90.0 (68 - 99.0)	
	Missing	0	0	-	

*Calculated using the Chi-square test

Table 2g Prevalence of SARS-CoV-2 seropositivity for general support staff, by role and hospital, PRECISE 2, April 2021

**Role**	**Hospital 1**			**Hospital 2**			**Both hospitals**		
	**Total**	**Seropositive**		**Total**	**Seropositive**		**Total**	**Seropositive**	
	**N**	**n**	**%**	**N**	**n**	**%**	**N**	**n**	**%**
Domestic/ Cleaning	62	15	24%	45	9	20%	107	24	22%
Catering	83	23	28%	18	5	28%	101	28	28%
Maintenance	27	6	22%	24	1	4.2%	51	7	14%
Security	33	6	18%	14	3	21%	47	9	19%
Porter	20	6	30%	13	1	7.7%	33	7	21%
Chaplain	11	3	27%	2	0	0.0%	13	3	23%
Other	7	1	14%	0	0	-	7	1	14%
Driver	0	0	-	6	2	33%	6	2	33%
Total	**243**	**60**	**25%**	**122**	**21**	17%	**365**	**81**	**22%**

Table 2h Prevalence of asymptomatic SARS-CoV-2 infection, by hospital location and ethnicity, April 2021

		Total seropositive	Asymptomatic SARS-CoV-2 infection		
		**N**	**n**	**% (95% CI)**	**p-value***
**St James's Hospital**	Total	623	121	19 (16 - 23)	0.009
	Irish	401	82	20 (17 - 25)	
	Other white background	56	13	23 (13 - 36)	
	Asian	122	13	11 (5.8 - 18)	
	African or other black background	29	11	38 (21 - 58)	
	Other	15	2	13 (1.7 - 40)	
**Galway University Hospital**	Total	275	48	17 (13 - 22)	0.531
	Irish	194	35	18 (13 - 24)	
	Other white background	38	5	13 (4.4 - 28)	
	Asian	26	5	19 (6.6 - 39)	
	African or other black background	10	3	30 (6.7 - 65)	
	Other	7	0	-	
**Both hospitals**	Total	898	169	19 (16 - 22)	0.010
	Irish	595	117	20 (17 - 23)	
	Other white background	94	18	19 (12 - 29)	
	Asian	148	18	12 (7.4 - 19)	
	African or other black background	39	14	36 (21 - 53)	
	Other	22	2	9.1 (1.1 - 29)	

*Calculated using the Chi-square test

Table 2i Undiagnosed SARS-CoV-2 infection, by HCW role and hospital location, April 2021

	St James's Hospital		University Hospital Galway		Both hospitals	
	n	%	n	%	n	%
**Admin**	19	10%	5	11%	24	10%
**Medical/dental**	12	6.3%	8	18%	20	8.5%
**Nursing/ midwifery**	79	42%	19	42%	98	42%
**Allied health**	32	17%	6	13%	38	16%
**General support**	22	12%	4	8.8%	26	11%
**Health care assistant**	26	14%	2	4.4%	28	12%
**Other**	0	0.0%	1	2.2%	1	0.4%
**Total**	190	100%	45	100%	235	100%

Table 3b Association between risk factors and SARS-CoV-2 seropositivity, Hospital 1, April 2021

Participant characteristics		Unadjusted relative risk (95% CI)	P-value	Adjusted relative risk (95% CI)	P-value
**Age groups (years)**	18-29	1.2 (0.9 - 1.4)	0.174	1.2 (1.0 - 1.5)	0.107
	30-39	1.0 (0.8 - 1.2)	0.677	1.0 (0.8 - 1.3)	0.909
	40-49	0.9 (0.7 - 1.1)	0.437	1.0 (0.8 - 1.2)	0.887
	50-59	Ref.		Ref.	
	Over 60	1.1 (0.8 - 1.5)	0.697	1.0 (0.8 - 1.5)	0.655
**Sex**	Female	Ref.		Ref.	
	Male	1.1 (0.9 - 1.3)	0.237	1.2 (1.0 - 1.4)	0.104
**Ethnicity**	Irish	Ref.		Ref.	
	Any other white background	1.1 (0.9 - 1.5)	0.312	1.1 (0.8 - 1.4)	0.516
	Asian background	1.4 (1.1 - 1.6)	0.001	1.1 (0.9 - 1.3)	0.571
	African or other black background	**2.2 (1.6 - 2.9)**	**<0.001**	**1.8 (1.3 - 2.4)**	**<0.001**

	Other	1.3 (0.9 - 2.1)	0.187	1.3 (0.8 - 2.0)	0.249
**Country of birth**	Ireland	Ref.			not entered
	India	1.4 (1.1 - 1.8)	0.005		
	Philippines	1.4 (1.1 - 1.8)	0.007		
	United Kingdom	1.0 (0.7 - 1.4)	0.908		
	Poland	1.6 (0.9 - 2.9)	0.108		
	USA	1.0 (0.4 - 2.4)	0.999		
	Romania	1.6 (1.0 - 2.5)	0.065		
	Nigeria	3.8 (2.9 - 4.9)	<0.001		
	Other	1.2 (0.9 - 1.5)	0.162		
**Education**	Primary	2.1 (1.1 - 3.8)	0.020	1.6 (0.9 - 2.9)	0.115
	Secondary	**1.5 (1.2 - 1.9)**	**<0.001**	**1.5 (1.2 - 1.9)**	**0.002**
	Third level	1.4 (1.2 - 1.6)	<0.001	1.2 (1.0 - 1.4)	0.103
	Post-graduate	Ref.		Ref.	
**Role**	Admin	Ref.		Ref.	
	Doctor\Dental	1.0 (0.7 - 1.4)	0.857	0.9 (0.6 - 1.3)	0.673
	Nursing	**1.6 (1.3 - 2.1)**	**<0.001**	**1.5 (1.1 - 2.0)**	**0.008**
	HCA	**2.5 (1.8 - 3.3)**	**<0.001**	**1.9 (1.4 - 2.6)**	**<0.001**
	General support	1.6 (1.1 - 2.1)	0.006	1.3 (0.9 - 1.8)	0.152
	Allied HCW	0.9 (0.7 - 1.2)	0.559	0.9 (0.7 - 1.3)	0.754
	Other	0.5 (0.2 - 1.2)	0.123	0.4 (0.2 - 1.1)	0.093
**Lives with**	Alone	Ref.			not entered
	With others	1.4 (1.0 - 1.9)	0.024		
**Lives with HCW**	No	Ref.		Ref.	
	Yes	**1.3 (1.1 - 1.5)**	**<0.001**	**1.2 (1.0 - 1.4)**	**0.011**
**Workplace exposure to COVID-19 patients**	No patient contact	Ref.		Ref.	
	Daily contact with patients without COVID-19	1.4 (1.1 - 1.7)	0.001	1.2 (0.9 - 1.4)	0.270
	Daily contact with COVID-19 patients	1.7 (1.4 - 2.1)	<0.001	1.2 (1.0 - 1.5)	0.090
**Previous COVID-19 like symptoms**	No	Ref.			not entered
	Yes	4.7 (3.9 - 5.7)	<0.001		
**Severity of symptoms**	No symptoms	Ref.			not entered
	Mild symptoms	3.2 (2.6 - 3.9)	<0.001		
	Significant symptoms	8.1 (6.7 - 9.8)	<0.001		

Severe symptoms (hospitalisation)	7.4 (4.9 - 11.2)	<0.001	

Table 3c Association between risk factors and SARS-CoV-2 seropositivity, Hospital 2, April 2021

**Participant characteristics**		**Unadjusted relative risk (95% CI)**	**P-value**	**Adjusted relative risk (95% CI)**	**P-value**
**Age groups (years)**	18-29	**2.0 (1.4 - 2.8)**	**<0.001**	**1.7 (1.2 - 2. 4)**	**0.004**
	30-39	1.5 (1.0 - 2.1)	0.034	1.3 (0.9 - 1.9)	0.120
	40-49	0.8 (0.6 - 1.2)	0.380	0.8 (0.5 - 1.2)	0.304
	50-59	Ref.		Ref.	
	Over 60	0.8 (0.4 - 1.6)	0.570	0.8 (0.4 - 1.5)	0.501
**Sex**	Female	Ref.		Ref.	
	Male	1.4 (1.1 - 1.8)	0.004	1.3 (1.0 - 1.7)	0.097
**Ethnicity**	Irish	Ref.		Ref.	

	Any other white background	1.5 (1.1 - 2.1)	0.009	1.3 (1.0 - 1.7)	0.078
	Asian background	1.8 (1.2 - 2.6)	0.002	1.2 (0.8 - 1.8)	0.333
	African or other black background	1.8 (1.0 - 3.2)	0.036	1.3 (0.7 - 2.4)	0.388
	Other	1.7 (0.8 - 3.3)	0.142	1.4 (0.7 - 2.8)	0.328
**Country of birth**	Ireland	Ref.			not entered
	India	2.1 (1.3 - 3.3)	0.001		
	Philippines	0.6 (0.1 - 3.7)	0.543		
	United Kingdom	1.0 (0.7 - 1.6)	0.841		
	Poland	1.8 (1.1 - 3.0)	0.027		
	USA	1.8 (0.9 - 3.6)	0.080		
	Romania	5.3 (2.6 - 11)	<0.001		
	Nigeria	0.9 (0.1 - 5.7)	0.899		
	Other	1.7 (1.2 - 2.4)	0.001		
**Education**	Primary	-			not entered
	Secondary	1.2 (0.8 - 1.8)	0.361		
	Third level	1.2 (0.9 - 1.5)	0.168		
	Post-graduate				
**Role**	Admin	Ref.		Ref.	
	Doctor\Dental	2.2 (1.4 - 3.6)	0.001	0.9 (0.5 - 1.5)	0.660
	Nursing	1.9 (1.2 - 2.9)	0.006	1.0 (0.6 - 1.6)	0. 901
	HCA	2.7 (1.5 - 4.6)	<0.001	1.5 (0.8 - 2.7)	0.180
	General support	2.2 (1.3 - 3.9)	0.005	1.1 (0.6 - 2.1)	0.704
	Allied HCW	**0.9 (0.5 - 1.5)**	**0.622**	**0.6 (0.3 - 1.0)**	**0.053**
	Other	1.5 (0.6 - 3.6)	0.331	0.8 (0.3 - 1.8)	0.533
**Lives with**	Alone	Ref.			not entered
	With others	1.3 (0.9 - 2.1)	0.188		
**Lives with HCW**	No	Ref.		Ref.	
	Yes	1.5 (1.2 - 1.8)	0.001	1.1 (0.9 - 1.4)	0.317
**Workplace exposure to COVID-19 patients**	No patient contact	Ref.		Ref.	
	Daily contact with patients without COVID-19	**2.5 (1.7 - 3.5)**	**<0.001**	**1.9 (1.3 - 2.9)**	**0.001**
	Daily contact with COVID-19 patients	**2.8 (1.9 - 4.1)**	**<0.001**	**2.1 (1.4 - 3.3)**	**0.001**
**Previous COVID-19 like symptoms**	No	Ref.			not entered
	Yes	8 (5.9 - 10.7)	<0.001		

**Severity of symptoms**	No symptoms	Ref.		not entered
	Mild symptoms	5.2 (3.8 - 7.3)	<0.001	
	Significant symptoms	14.2 (10.4 - 19.3)	<0.001	
	Severe symptoms (hospitalisation)	19.4 (13.3 - 28.2)	<0.001	

Table F. Characteristics of fully vaccinated participants with PCR- confirmed infection i.e. vaccine breakthrough cases, both hospitals (n=23), PRECISE 2, April 2021

Participant characteristics		**PCR positive ≥14 days after second vaccine dose (N=23)**	
		**n**	**%**
**Hospital**	Hospital 1	18	78%
	Hospital 2	5	22%
**Age groups**	18-29	2	8.7%
	30-39	6	26%
	40-49	8	35%
	50-59	5	22%
	≥60	2	8.7%
**Sex**	Female	15	65%
	Male	8	35%
**Ethnicity**	Irish	12	52%
	Any other white background	1	4.3%
	Asian background	8	35%
	African or other black background	2	8.7%
	Other	0	0.0%
**Country of birth**	Ireland	12	52%

	United Kingdom	0	0.0%
	India	4	17%
	Philippines	4	17%
	Poland	1	4.3%
	USA	0	0.0%
	Other	0	0.0%
**Education**	Primary	0	0.0%
	Secondary	2	8.7%
	Third level	11	48%
	Post-graduate	10	43%
**Role**	Admin	0	0.0%
	Medical/dental	0	0.0%
	Nursing/ midwifery	10	43%
	Allied health	5	22%
	General support	2	8.7%
	Health care assistant	4	17%
	Other	2	8.7%
**Workplace exposure to COVID-19 patients**	No patient contact	2	8.7%
	Daily contact with COVID-19 patients	9	39%
	Daily contact with patients without COVID-19	12	52%
**Lives with**	Alone	1	4.3%
	With others	22	96%
	Missing	0	0.0%
**Lives with HCW**	Yes	13	57%
	No	10	43%
**Vaccine type**	Pfizer	23	100%
	Other	0	0.0%
**Symptoms at the time of positive PCR test**	Yes	5	22%
	No	18	78%

Table 4 Comparison of SARS-CoV-2 seroprevalence October 2020 and April 2021, by hospital

Participant characteristics		Hospital 1			Hospital 2			Both hospitals		
		%	%	%	%	%	%	%	%	%
		Oct-20	Apr-21	change	Oct-20	Apr-21	change	Oct-20	Apr-21	change
**Overall seroprevalence**		15	21	6.2	4.1	13	8.8	10	18	7.7
**Age groups (years)**	18-29	20	24	4.3	4.7	20	15.1	13	22	9.5
	30-39	15	20	5.1	6	15	8.9	10	18	7.9
	40-49	13	19	6.4	3.5	8.5	5.0	8.2	15	6.5
	50-59	13	21	8.1	1.5	10	8.6	7.7	17	8.9
	Over 60	17	23	5.5	2.7	8.4	5.7	9.9	16	6.1
**Sex**	Female	15	21	5.7	3.5	12	8.3	9.4	17	7.5
	Male	16	23	6.8	6.3	17	10.5	12	20	8.3
**Ethnicity**	Irish	13	19	6.2	3.7	11	7.7	8.6	16	7.1
	Any other white background	17	22	4.8	6.3	17	11.1	11	20	8.7
	Asian background	24	26	2.0	7.1	20	13.1	19	25	5.7
	African or any other black background	23	42	19	2.1	21	18.7	14	33	19
	Other	13	26	13	-	19	-	6.9	23	16
**Country of birth**	Ireland	13	19	6.1	3.9	11	7.4	8.7	16	6.9
	United Kingdom	16	19	2.7	3.7	12	8.1	9.3	15	5.6
	India	23	27	3.7	8.2	24	15.3	18	26	7.9
	Philippines	27	27	-0.2	8.0	6.3	-1.8	25	25	0.2
	Poland	29	31	1.8	6.3	20	14	14	24	9.5
	USA	14	19	5.0	-	21	-	5.0	20	15
	Other	16	28	12	4	28	23.5	10	25	15
**Education**	Primary	15	35	20	0.0	0.0	0.0	14	32	18
	Secondary	13	26	13	3.0	14	11	8.9	22	13
	Third level	18	24	5.5	4.3	14	9.5	11	19	8.3
	Post-graduate	14	17	3.0	4.1	12	7.6	9.0	15	5.7
**Role**	Admin	10	16	5.9	1.2	7.7	6.5	6.0	13	6.6
	Medical/dental	14	15	1.4	6.9	17	10.2	10	16	6.3

	Nursing/ midwifery	21	26	4.6	4.7	14	9.7	13	21	7.9
	Allied health	10	15	4.5	2.5	6.7	4.2	6.7	11	4.6
	General support	12	25	13	1.7	17	15.5	7.6	22	15
	Health care assistant	27	39	12	6.2	21	14.3	18	32	14
	Other	11	7.4	-3.6	1.4	12	10.4	5.5	9.5	4.0
**Lives with**	Alone	8.2	16	7.4	3.1	10	6.7	5.9	13	7.2
	With others	16	22	5.7	4.2	13	9.0	10	18	8.1
	Missing	11	38	27	-	-	-	9.1	27	18
**Lives with HCW**	Yes	21	25	4.2	4.9	16	11.6	13	22	8.6
	No	13	19	6.0	3.8	11	7.5	8.5	16	7.3
	Missing	17	25	8.0	2.1	10	8.1	9.9	18	7.7
**Daily contact with COVID-19 patients**	Contact with COVID-19 patients	21	27	6.0	7.1	17	9.7	15	23	8.3
	Contact with patients without COVID-19	17	22	4.5	4.6	15	10.3	11	19	7.5
	No patient contact	9.5	16	6.1	1.3	6.1	4.8	5.9	12	5.8
**Previous COVID-19 symptoms**	No symptoms	5.8	7.7	1.9	1.3	3.6	2.3	3.2	5.8	2.6
	Had symptoms	23	37	14	7.5	28	21	17	34	17
**Previous positive COVID-19 PCR test**	No	6.8	7.8	1.0	1.5	2.4	0.9	4.2	5.5	1.3
	Yes	94.9	84	-11	97.3	78	-19.1	95.4	82	-14
